# Accelerated Biological Aging in Exfoliation Glaucoma Assessed by Fundus-Derived Predicted Age and Advanced Glycation End Products

**DOI:** 10.3390/ijms26104725

**Published:** 2025-05-15

**Authors:** Masaki Tanito, Makoto Koyama

**Affiliations:** 1 Department of Ophthalmology, Faculty of Medicine, Shimane University, Enya 89-1, Izumo 693-8501, Shimane, Japan; 2 Minamikoyasu Eye Clinic, 2-8-30 Minamikoyasu, Kimitsu 299-1162, Chiba, Japan; minamikoyasuganka@gmail.com

**Keywords:** exfoliation glaucoma, biological aging, fundus photographs, predicted age acceleration, advanced glycation end products, biomarker, oculomics

## Abstract

Glaucoma is an age-related neurodegenerative disease characterized by progressive optic nerve damage. Accelerated biological aging, assessed using predicted age derived from fundus images, may serve as a biomarker for glaucoma progression. This study aimed to examine fundus-derived age acceleration among patients with primary open-angle glaucoma (POAG), exfoliation glaucoma (EXG), and controls, and to explore its biochemical basis through advanced glycation end products (AGEs). Fundus photographs from 237 participants (79 POAG, 79 EXG, and 79 age- and sex-matched controls) were analyzed using a deep learning model (EfficientNet) previously trained to predict biological age. AGE accumulation was assessed by measuring skin autofluorescence (sAF). Multivariate regression analyses were conducted to identify factors influencing predicted age acceleration, with stratification into age tertiles to control for age-related effects. EXG patients demonstrated significant accelerated biological aging compared to controls (*p* = 0.006), particularly evident in younger and middle-aged tertiles. AGE scores were significantly elevated in EXG relative to both POAG (*p* = 0.009) and control groups (*p* = 0.003). Predicted age and AGE scores were more strongly correlated than chronological age and AGEs, especially in the middle tertile (*p* = 0.002). Accelerated biological aging detected via fundus images occurs prominently in EXG, potentially reflecting underlying AGE accumulation. Fundus-derived predicted age could serve as a non-invasive biomarker for assessing glaucoma progression risk and warrants further exploration in clinical applications.

## 1. Introduction

Glaucoma, affecting an estimated 3.54% of individuals aged between 40 and 80 years globally [1], remains a leading cause of severe visual impairment and blindness worldwide [2,3]. Glaucomatous optic neuropathy results primarily from the degeneration of retinal ganglion cells (RGCs) and the subsequent loss of RGC axons [4]. Elevated intraocular pressure (IOP) is recognized as the primary risk factor for open-angle glaucoma (OAG), encompassing both primary open-angle glaucoma (POAG) and exfoliation glaucoma (EXG) secondary to pseudoexfoliation syndrome [4]. The rise in IOP in these glaucoma types results from impaired aqueous humor drainage from the anterior chamber into Schlemm’s canal via the trabecular meshwork (TM)–Schlemm’s canal endothelium complex [5]. In POAG, increased resistance within the TM is attributed to alterations in the quantity and quality of the extracellular matrix (ECM) in the trabecular meshwork [6]. EXG, characterized as an age-related generalized disorder of ECM metabolism, involves the progressive accumulation of abnormal fibrillar deposits within the TM, leading to sustained elevation of IOP [7,8]. Various genetic, internal, and environmental stressors, including immune responses, inflammatory processes, ischemia, hypoxia, and oxidative stress, have been implicated in increasing TM resistance and subsequent RGC death [9,10,11]. Although IOP remains the primary modifiable risk factor for glaucoma, both myopia and advanced age have been identified as significant risk factors for its development [12]. The Tajimi Study, a major population-based investigation conducted in Japan, emphasized these associations, highlighting the necessity for comprehensive glaucoma risk evaluations [13]. Furthermore, population-based studies such as the Barbados Eye Studies have underscored older age as a critical factor influencing the onset and progression of glaucoma [14].

Recent advancements have shown that deep learning algorithms trained on fundus images can effectively diagnose glaucoma with high accuracy, sensitivity, and specificity [15,16]. These technological developments demonstrate significant potential for enhancing glaucoma screening and diagnosis. Advancements in imaging technologies and machine learning have given rise to the field of oculomics, which investigates ocular biomarkers as indicators of systemic health and disease risks [17,18,19,20]. Oculomics has shown promise in correlating ocular features, particularly vascular alterations in the fundus, with systemic conditions such as cardiovascular diseases, neurodegenerative disorders, and diabetes [17,18,19,20,21]. The incorporation of deep learning techniques into oculomics has significantly improved its capabilities, allowing detailed analyses of fundus images for evaluating both ocular and systemic health conditions [22,23]. Among recent breakthroughs, fundus-derived age prediction, an innovative biomarker generated through deep learning methodologies, has become an attractive approach for assessing ocular and systemic health statuses. Differences between predicted and chronological age, termed “predicted age acceleration” or “retinal age gap”, might reflect underlying pathological conditions, including systemic age-related disorders such as stroke and increased mortality risk [24,25] and ocular diseases like diabetic retinopathy and glaucoma [26,27]. Thus, fundus-based age estimation has considerable potential as a biological clock. Recently, we reported that, within a general health-check setting, glaucomatous eyes exhibited older predicted fundus ages compared to control eyes [28]. However, the relationship between glaucoma subtype and accelerated biological aging, as indicated by fundus-derived age prediction, remains unclear.

Advanced glycation end products (AGEs) represent a diverse group of lipids, proteins, and nucleic acids undergoing glycation resulting from prolonged exposure to glucose or other reducing sugars. AGEs accumulate primarily in long-lived proteins such as collagen, contributing to oxidative stress within tissues [29,30,31]. Since the late 1990s, AGEs have been assessed non-invasively using the AGE Reader (DiagnOptics Technologies B.V., Groningen, The Netherlands), which uses a light-emitting diode emitting wavelengths from 300 to 420 nm to measure skin autofluorescence (sAF) within the 420 to 600 nm range [32,33,34]. Enzyme-linked immunosorbent assays conducted on diabetic patients revealed that specific AGEs accumulate in skin tissue before the clinical onset of microvascular complications [35]. The formation and accumulation of AGEs have been linked to metabolic disorders, notably diabetes mellitus [36,37,38], as well as physiological aging processes [29,30,31,39,40]. Consequently, AGE detection serves as a potential biomarker for identifying abnormal aging processes [41,42,43,44,45,46]. Prior research suggests that AGEs contribute to the onset and progression of chronic age-related conditions such as cardiovascular disease, heart failure, chronic kidney disease, dementia, and other neurodegenerative diseases [36,47,48,49]. Furthermore, AGEs have been implicated in the pathophysiology of various ocular diseases, including cataract, diabetic retinopathy, and age-related macular degeneration [50,51,52]. Glaucoma, an age-related chronic neurodegenerative disorder significantly influenced by oxidative stress, has also been associated with AGE accumulation, notably in the optic nerve head and retinal ganglion cell (RGC) layers [53,54,55,56]. Thus, AGEs represent a promising biomarker for abnormal aging processes specifically associated with glaucoma.

This study aimed to elucidate the relationship between glaucoma and accelerated biological aging by comparing fundus-derived predicted ages among three groups: POAG and EXG, which are both representative types of OAG, and a non-glaucomatous control group. Additionally, the study examined the association between age-related parameters and AGE scores to evaluate the effectiveness and utility of fundus-derived predicted age as a biomarker for aging.

## 2. Results

Comparisons of background parameters among the control, POAG, and EXG groups are summarized in Table 1. Each group included 79 eyes from 79 subjects. There were no significant intergroup differences in sex distribution, body-mass index (BMI), systolic blood pressure (SBP), diastolic blood pressure (DBP), heart rate (HR), prevalence of diabetes, or best-corrected visual acuity (BCVA). The subjective spherical equivalent refractive error (SERE) was significantly more myopic in the POAG group compared to controls (*p* = 0.004). Lens status revealed a significantly higher proportion of eyes with intraocular lens implantation (IOL) in both the POAG (*p* < 0.0001) and EXG (*p* < 0.0001) groups compared to the control group. Intraocular pressure (IOP) was significantly elevated in the POAG (*p* < 0.0001) and EXG (*p* < 0.0001) groups compared with controls, with the EXG group having significantly higher IOP than the POAG group (*p* < 0.0001).

The comparison of true age, predicted age, and prediction difference among the control, POAG, and EXG groups are summarized in Table 2. True age did not differ significantly among the three groups (*p* = 0.84). However, predicted age significantly varied across groups (*p* = 0.0005). Pairwise group comparisons revealed no difference between the control and POAG groups (*p* = 0.82), but the EXG group showed significantly older predicted age compared to the control group (*p* = 0.0008) and the POAG group (*p* = 0.006). Consequently, the prediction difference similarly exhibited no significant difference between the control and POAG groups (*p* = 0.67), whereas the EXG group had a significantly higher prediction difference compared to the control group (*p* = 0.006). The difference between the EXG and POAG groups was not significant (*p* = 0.06).

Multivariate analysis was performed to identify factors associated with the prediction difference (Table 3). Older true age was significantly associated with younger predicted age (*p* < 0.0001), while eyes with IOL were predicted to be significantly older (*p* = 0.01). Sex, BMI, SBP, DBP, HR, diabetes status, BCVA, SERE, and IOP did not significantly influence age prediction. Even after adjusting for these covariates, EXG remained significantly associated with higher predicted age compared to controls (*p* = 0.03), whereas POAG did not differ significantly from the control group (*p* = 0.70). Variance inflation factors (VIFs) for all variables were below 3.5, indicating an absence of significant multicollinearity. These findings suggest that fundus age is predicted to be higher in EXG compared to controls.

**Figure 1:**
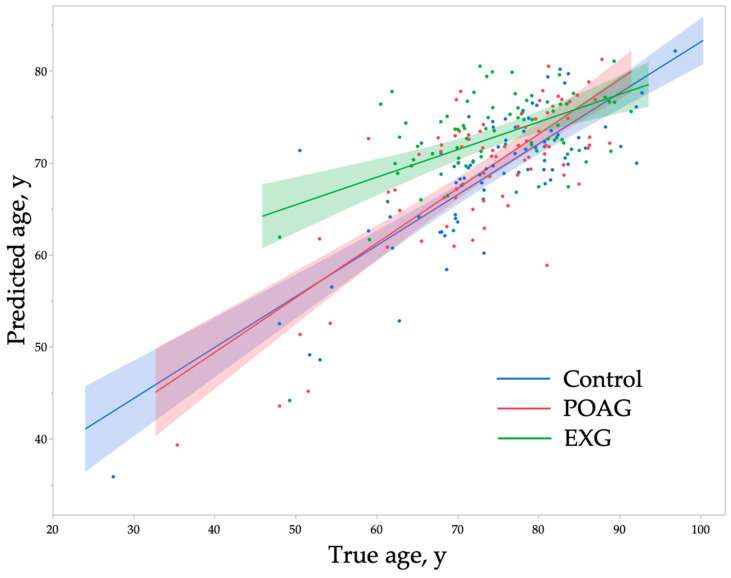
The relationship between true age and predicted age. The line represents the linear regression line for each group, and the shaded area indicates the 95% confidence interval. POAG, primary open-angle glaucoma; EXG, exfoliation glaucoma.

Figure 1 shows the relationship between true age and predicted age across the control, POAG, and EXG groups. Examination of the regression lines with shaded 95% confidence intervals (CIs) indicates overlap among the three groups in subjects aged 75 years or older; however, in younger age groups, the regression line for EXG shifts upwards, indicating higher predicted ages. To evaluate the influence of true age on age prediction, subjects were categorized into tertiles based on true age. Table 4 presents a comparison of predicted age and prediction difference among the three groups within each age tertile. In T1 (≤70.8 years), significant differences among the three groups were found in predicted age (*p* = 0.005) and prediction difference (*p* = 0.006), with the highest predicted ages in the EXG group, followed by the POAG group, and then the control group. Similarly, in T2 (70.8–79.2 years), predicted age (*p* < 0.0001) and prediction difference (*p* < 0.0001) differed significantly among groups, with EXG predicted to be significantly older compared to the other two groups. Conversely, in T3 (>79.2 years), there were no significant differences among the groups in either predicted age (*p* = 0.86) or prediction difference (*p* = 0.58). These findings suggest that the accelerated age prediction associated with EXG predominantly occurs before advanced age.

**Figure 2:**
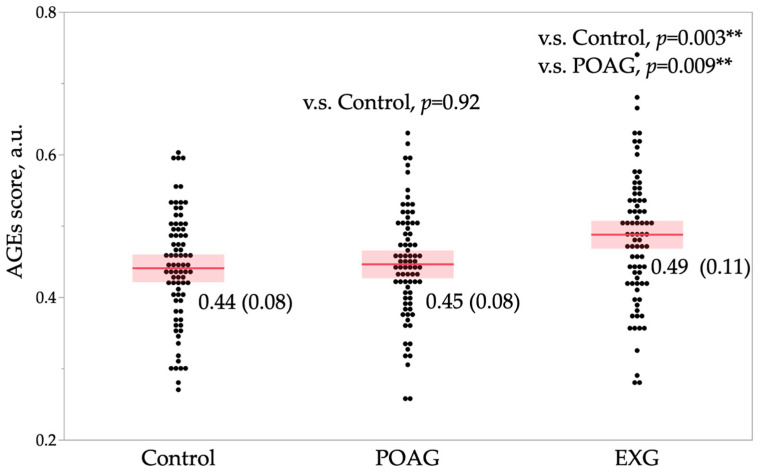
A comparison of AGE scores among the three groups. The *p*-value for the comparison among the three groups, calculated using one-way analysis of variance, is 0.001. The *p*-values shown within the graph were calculated using post-hoc Tukey’s HSD test for each pairwise comparison. Data are expressed as mean (standard deviation). The red line represents the mean, and the shaded area indicates the 95% confidence interval. The double asterisks (**) indicate *p* < 0.01. AGE, advanced glycation end products; a.u., arbitrary units; POAG, primary open-angle glaucoma; EXG, exfoliation glaucoma.

**Figure 3:**
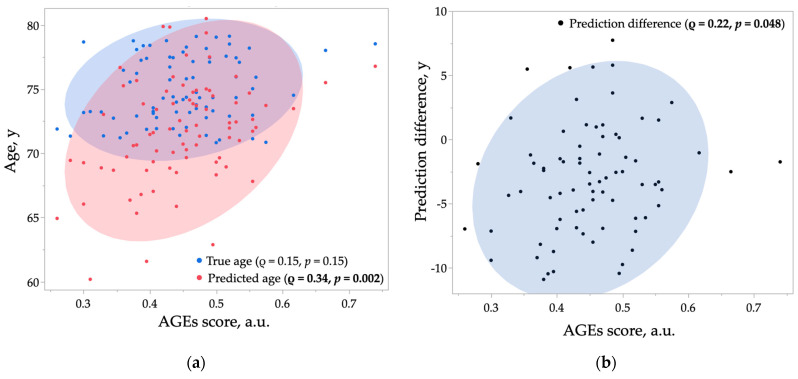
Scatter plots showing the relationship between AGE score and age (**a**) and prediction difference (**b**) in T2 (70.8–79.2 years). The ρ and *p* values were obtained by Spearman’s rank test. The shaded areas represent 90% probability ellipses. AGE, advanced glycation end products; a.u., arbitrary units.

Figure 2 presents the comparison of AGE scores among the control, POAG, and EXG groups. AGE scores differed significantly across the three groups (*p* = 0.001). Post-hoc analysis revealed significantly higher AGE scores in the EXG group compared to both the control (*p* = 0.003) and POAG (*p* = 0.009) groups. Table 5 shows correlations between AGE scores and age-related parameters within each tertile. True age did not correlate significantly with AGE scores in any of the tertiles (T1, T2, T3). However, predicted age (*p* = 0.002) and prediction difference (*p* = 0.048) exhibited significant positive correlations with AGE scores in the T2 tertile. Figure 3 illustrates the distribution of AGE scores and age parameters within the T2 tertile. Predicted age (Figure 3a, shaded red area) correlated more closely with changes in AGE score compared to true age (Figure 3a, shaded blue area). Similarly, prediction difference showed a correlation with AGE score changes (Figure 3b, shaded blue area). These findings suggest that predicted age may more accurately reflect tissue AGE accumulation compared to true age, particularly within certain age ranges.

**Figure 4:**
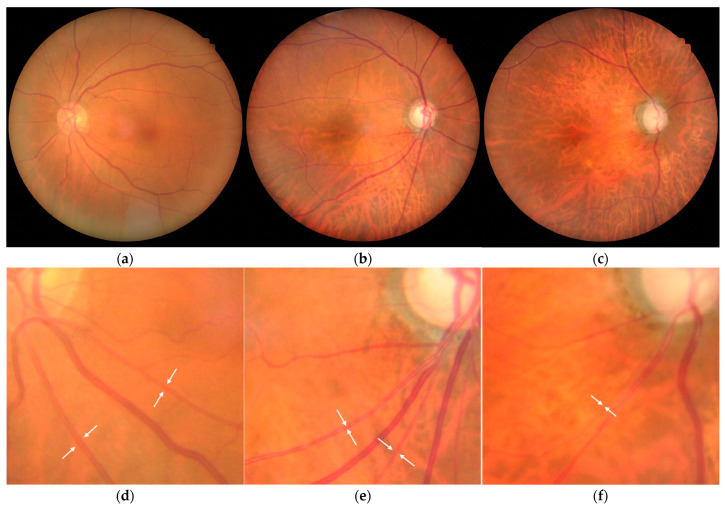
Representative fundus photographs. (**a**,**d**) Control, female, pseudophakia, left eye. True age = 70.02, estimated age = 63.56, prediction difference = −6.46. (**b**,**e**) POAG, female, phakia, right eye. True age = 72.79, estimated age = 73.42, prediction difference = +0.63. (**c**,**f**) EXG, female, pseudophakia, right eye. True age = 71.21, estimated age = 76.67, prediction difference = +5.48. Arrows indicate retinal arterioles.

Finally, representative fundus photographs are displayed in Figure 4 (a, d: control; b, e: POAG; c, f: EXG). In these examples, the prediction differences were −6.46 years for the control, +0.63 years for POAG, and +5.48 years for EXG. A visual comparison reveals that in addition to glaucomatous changes in the optic disc, the fundus background appears brighter in glaucoma groups. Additionally, noticeable retinal arterial narrowing is observed in the glaucoma groups (d, e, f, arrows).

## 3. Discussion

This study demonstrated accelerated biological aging assessed through fundus photographs in patients with EXG. Additionally, predicted age derived from fundus images showed a stronger correlation with AGE scores compared to chronological age, except in very elderly participants. Previous studies have suggested the involvement of systemic aging processes in the onset and progression of glaucoma [57,58]. Our findings specifically suggest a close link between systemic aging and glaucoma-related ocular aging, particularly in EXG.

Interestingly, the prediction differences were negative across the control, POAG, and EXG groups (Table 2), suggesting the model consistently predicted younger ages than actual chronological ages. This observation likely arises from the characteristics of the prediction model employed [28]. The model was trained on a cohort with an average age of 64.6 years, whereas the current study’s subjects had an older mean age of approximately 74 years. Consequently, the deep learning algorithm may have systematically underestimated ages in older individuals due to the underrepresentation of older age groups in the training dataset. This potential bias aligns with the negative correlation observed between true age and prediction difference in multivariate analysis (Table 3). However, several methodological steps were implemented to ensure that the differences in predicted age were specifically attributable to glaucoma rather than age-related biases. Firstly, the three study groups were precisely age-matched using propensity score matching. Secondly, the impact of chronological age was thoroughly controlled through multivariate analyses, confirming EXG as an independent factor associated with accelerated predicted age after adjusting for age effects (Table 3). Thirdly, analyses were stratified into tertiles based on chronological age to further control for potential age-related confounding (Table 4). These methodological precautions substantiate the conclusion that the differences observed reflect genuine biological aging processes linked with EXG.

Analysis by age tertiles demonstrated age acceleration only within the T1 and T2 groups (Table 3). This observation aligns with the distributions of true and predicted ages by group shown in Figure 1. The presence of pseudoexfoliation material typically occurs in individuals aged 50 years or older, and its prevalence approximately doubles in individuals in their 80s compared to those in their 70s [59]. The prevalence of pseudoexfoliation syndrome has been reported to be 2.5% (pseudoexfoliation syndrome and exfoliation glaucoma combined) among individuals aged 70 years and older in the Tajimi study [59] and 3.4% among individuals aged 50 years or older in the Hisayama study [60]. Thus, the age acceleration observed in our study may represent an event occurring relatively early in the clinical progression of EXG. EXG frequently results in drug-resistant elevated IOP. According to a glaucoma surgery patient survey, EXG accounts for 18% of all glaucoma surgeries, with this proportion increasing from 17% among those aged 65–74 years to 26% among individuals aged 75–89 years, and further to 33% among those aged 90 years or older [61]. Although pseudoexfoliation material deposition is initially unilateral in more than half of cases, about half of these patients develop bilateral involvement within 15 years, and approximately 30% of individuals with pseudoexfoliation syndrome eventually develop EXG [62]. The Early Manifest Glaucoma Trial reported that the presence of pseudoexfoliation material doubled the risk of progression in OAG [63], with notably faster progression rates compared to normal-tension glaucoma (−0.36 dB/year) and high-tension glaucoma (−1.31 dB/year), at −3.13 dB/year [64]. Additionally, untreated baseline IOP in patients with EXG increases annually by approximately 0.96 mmHg, while it remains stable in POAG [65]. Given the severity and rapid progression associated with EXG, proactive clinical management strategies are particularly essential in aging societies. Further research is needed to clarify how the detection of accelerated biological aging through fundus photography could aid in predicting the onset and progression of EXG. In our previous analysis using a dataset from participants of the health checkup program, we reported that fundus age acceleration was observed in elderly individuals (≥79 years) with glaucoma [28]. Given that these participants represent the general population, the majority of glaucoma cases in that study were likely POAG [59]. In contrast, the present hospital-based study adjusted the number of cases in each group to a 1:1:1 ratio. Therefore, our previous findings likely reflect age-related acceleration primarily in POAG. Indeed, in the current analysis, although not statistically significant, the predicted age in the POAG group tended to be higher in the older age group (Figure 1, Table 4, T3). These findings suggest that the phase of fundus age acceleration may differ between POAG and EXG.

The mechanisms underlying accelerated aging detected through fundus photographs remain unclear. In this study, we employed AGE scores as markers of biological change. Hondur et al. previously reported significantly elevated AGE levels in both aqueous humor and blood samples from glaucoma patients compared to non-glaucoma controls [54]. Experimental findings also suggest that AGEs and their receptors accumulate in glaucoma-associated tissues, such as the optic nerve head, RGC layer, and surrounding vasculature, potentially contributing to glaucomatous optic neuropathy [53,56]. Multiple studies have investigated AGE accumulation in glaucoma patients [33,66]. Schweitzer et al. demonstrated a higher prevalence of OAG (not limited to POAG) in individuals with elevated sAF, indicating higher AGE levels compared to subjects with normal AGEs [33]. Our previous studies specifically reported increased AGE scores in patients with EXG but not in those with POAG [67,68]. Therefore, the current findings (Figure 2) consistently replicate our earlier results.

Specific AGEs, such as CML and pentosidine, can result from both glycation (Maillard reaction) and oxidative processes [69]. Notably, CML accumulation has been observed in the lens capsules of individuals with pseudoexfoliation syndrome [70]. Although systemic antioxidant capacity is reportedly diminished in both POAG and EXG compared to controls [71,72,73,74], our prior research indicated distinct oxidative mechanisms between POAG and EXG. Comprehensive analyses of serum hydroxylinoleate isomers have revealed enzymatic and singlet oxygen-mediated fatty acid oxidation as predominant in POAG [75,76,77], whereas EXG primarily involves free radical-mediated oxidation [78]. Reactive intermediates, such as α-dicarbonyls (including methylglyoxal (MGO) and 3-deoxyglucosone), form during the Amadori rearrangement [79]. MGO is generated through non-oxidative processes such as anaerobic glycolysis [80] and the oxidative degradation of polyunsaturated fatty acids [81]. In vitro experiments have shown antioxidants to reduce CML formation, indicating that non-enzymatic lipid oxidation significantly contributes to AGE generation [82,83]. Thus, variations in oxidative stress pathways between POAG and EXG may account for the observed elevation of AGEs specifically in EXG. Accelerated fundus aging observed in T1 and T2, along with the association with AGEs found specifically in T2, suggests that individuals in their 70s may be at a critical age at which radical-mediated oxidation and AGE accumulation surpass a threshold, potentially contributing to the onset of EXG. If accelerated aging and elevated AGE scores indeed influence the onset or progression of EXG, these factors may become potential intervention targets beyond intraocular pressure control. Dietary modifications or supplementation strategies aimed at reducing AGE accumulation could provide therapeutic benefits. Furthermore, AGE scores might serve not only as theoretical support for these interventions but also as biomarkers for evaluating treatment efficacy.

Pseudoexfoliation material consists of insoluble, white fibrillar structures composed of thin (10–15 nm) and thicker (20–40 nm) fibers, which aggregate into mature fibers approximately 50 nm in diameter, containing elastic fiber components such as elastin and fibrillin [84,85]. Potential sites of pseudoexfoliation material production include ocular tissues such as TM cells, lens epithelium, corneal endothelium, and ciliary epithelium, as well as systemic tissues like blood vessels and muscles [8,86]. In EXG, vascular changes leading to compromised ocular blood flow and connective tissue degeneration in the lamina cribrosa could contribute to glaucoma onset and progression. Previous analyses using stereo fundus photography have reported age-related narrowing of retinal vascular diameters, including central retinal arteriolar and venular equivalents, in glaucomatous eyes. These vascular parameters also correlated with glaucomatous optic nerve head changes [87]. Thus, the age acceleration detected through fundus photography may reflect underlying alterations in retinal vessels. Additionally, progressive visual field loss in EXG has been associated with notable retinal vascular narrowing, particularly of retinal arterioles [88]. Moreover, evaluation of fundus images from population health check-ups revealed strong associations between hypertensive and arteriosclerotic retinal vascular changes and glaucoma, as well as correlations with predicted age [28]. Quantitative assessments of retinal arteriolar narrowing from fundus images have further linked these changes with long-term glaucoma risk [89]. In the present study, fundus photographs frequently revealed prominent retinal vascular alterations alongside optic disc changes in glaucoma patients (Figure 4). These observations suggest that our age prediction model captures not only optic nerve head alterations but also age-related vascular and retinal changes. However, the exact image features recognized by our model remain unclear, representing an important area for future investigation.

Various aging hallmarks have been extensively described through both basic and clinical research [90]; however, a definitive unifying theory or key molecular mechanism underlying aging has yet to be identified. Epigenomic markers used to evaluate accelerated biological aging have demonstrated associations with multiple diseases [91]. In comparison, fundus-derived biological age prediction provides a notable advantage, as it is non-invasive and utilizes broadly accessible imaging technology, unlike more specialized epigenomic assessments. Fundus-based predicted age acceleration may represent a promising biomarker for detecting individuals at increased risk for glaucoma progression. Incorporating deep learning into fundus image analysis could enable clinicians to concurrently evaluate typical glaucoma-related structural changes and broader systemic health factors influencing disease trajectory. This methodology supports the expanding interest in precision medicine and the clinical adoption of oculomics.

While this study provides valuable insights, several limitations must be acknowledged. The cross-sectional study design prevents causal inference regarding the relationship between glaucoma and predicted age acceleration. Accordingly, the temporal dynamics of age acceleration—specifically, whether it precedes the onset of disease—remain unknown. Longitudinal studies are necessary to determine whether accelerated age prediction precedes or results from glaucoma progression. Glaucoma encompasses a variety of subtypes, and it is important to note that the present study is limited to POAG and EXG within the spectrum of open-angle glaucoma when interpreting the results. Although we evaluated systemic factors such as self-reported diabetes and blood pressure, data on other systemic comorbidities and systemic medications were unavailable. Despite excluding patients with systemic disease-related ocular conditions, such as diabetic retinopathy or retinal hemorrhages, from fundus image analysis, the influence of systemic comorbidities on age prediction cannot be entirely ruled out. Additionally, data on glaucoma severity, treatment history, and disease duration were missing, representing further limitations. We matched participants across glaucoma subtypes based on age and sex; however, significant differences among groups were noted for several background parameters (BCVA, SERE, lens status, IOP). Fundus coloration changes due to visible choroidal patterns in myopic eyes can influence age prediction outcomes. Given that the POAG group showed the most significant myopic shift and SERE was adjusted in multivariate analyses, the impact on the significant age acceleration observed in the EXG group is likely minimal. The multivariate analysis revealed a significant association between IOL implantation and accelerated age prediction. The higher prevalence of IOL eyes in glaucoma groups may have influenced the univariate analysis outcomes. However, adjustment in the multivariate analysis and the finding that significant differences were observed predominantly in younger tertiles rather than older tertiles with higher IOL prevalence support the validity of accelerated aging results in EXG. The fundus photographs used in this study and those used for developing the age prediction model were acquired with different fundus cameras, potentially affecting prediction accuracy. Nonetheless, deep learning models for fundus image interpretation have demonstrated robustness across images from different cameras [92]. Furthermore, the root mean square error (RMSE) for age prediction was 5.06 years in this study, which is comparable to the RMSE of 8.45 years observed previously in our test dataset (including glaucomatous eyes) [28], indicating no substantial reduction in accuracy. Additionally, the study population was drawn from a single geographic area, limiting the generalizability of findings to broader populations. The interpretability of the deep learning models employed remains limited, requiring further exploration of underlying biological mechanisms. Future research should validate these results across diverse populations and explore longitudinal relationships between predicted age acceleration and glaucoma progression. Although these findings are not immediately translatable to direct clinical practice, improved understanding of aging mechanisms may support the development of future glaucoma treatments.

## 4. Materials and Methods

### 4.1. Subjects

This study was conducted as a retrospective observational study. This research was conducted in accordance with the Declaration of Helsinki and the Ethical Guidelines for Life Science and Medical Research Involving Human Subjects issued by the Japanese government. Ethical approval was granted by the Medical Research Ethics Committee at Shimane University Faculty of Medicine (IRB No. KS20230719-3, latest approval: 22 April 2024; and K20200228-2, latest approval: 10 March 2025). Instead of obtaining written informed consent, the study details were publicly announced on the institutional website, offering participants an opt-out method.

Eligible cases were selected from the patient records at Shimane University’s Department of Ophthalmology based on specified inclusion and exclusion criteria. Inclusion criteria consisted of the following: patients who attended Shimane University Hospital’s ophthalmology department between June 2022 and July 2024; patients diagnosed with POAG or EXG in at least one eye, or those without ocular diseases affecting vision other than cataracts in both eyes; available AGE measurements obtained from fingertip skin; clear color fundus photographs captured for at least one eye using a nonmyd WX fundus camera (Kowa Company, Ltd., Aichi, Japan); documented blood pressure, height, and weight measurements; and recorded medical history concerning diabetes mellitus. Patients were excluded if they had vision-impacting anterior segment or fundus diseases apart from glaucoma and cataracts or previous intraocular surgeries other than those performed for glaucoma or cataracts. Additionally, cases of diabetic retinopathy were deliberately excluded due to the known association between AGEs and diabetic conditions or their related complications [93,94,95].

Date of birth, fundus photography date, sex, disease type, systolic blood pressure (SBP), diastolic blood pressure (DBP), heart rate (HR), presence or absence of diabetes mellitus, the highest IOP recorded, lens status, best-corrected visual acuity (BCVA), and subjective spherical equivalent refractive error (SERE) were collected through medical record reviews. All participants underwent IOP measurement using a Goldmann applanation tonometer, slit-lamp examination, and fundus examination. Glaucoma diagnosis was made according to the guidelines set by the Japan Glaucoma Society [96]. POAG was diagnosed based on open iridocorneal angles bilaterally, characteristic signs of glaucomatous optic neuropathy such as enlargement of the optic disc cup or focal thinning of the neuroretinal rim, corresponding visual field defects in at least one eye, and absence of secondary glaucoma signs. EXG diagnosis required open iridocorneal angles and characteristic pseudoexfoliation material deposition on the lens capsule or pupillary margin in at least one eye. In cases where both eyes met diagnostic criteria, the eye with a worse visual field mean deviation (MD) was selected for the POAG or EXG analysis. Visual fields were evaluated using automated perimetry (Humphrey Visual Field Analyzer, Carl Zeiss Meditec, Dublin, CA, USA). Control subjects had no significant ocular conditions except for age-related cataract, no clinical indications of glaucoma, and were not using glaucoma medications. In control subjects, the highest recorded IOP was <21 mmHg. The control eye chosen was the one with better BCVA; if BCVA was identical in both eyes, the right eye was selected. Consequently, a total of 510 eyes from 510 subjects were included in the analysis.

### 4.2. Propensity Score Matching

Propensity scores were generated using multinomial logistic regression, incorporating age and sex, to ensure balanced baseline characteristics across the POAG (N = 289), EXG (N = 142), and control (N = 79) groups. A greedy nearest-neighbor matching method was employed to form matched sets (1:1:1), taking the smallest group (control group, n = 79) as a reference. For each participant in the control group, propensity score vectors were calculated, and subjects from POAG and EXG groups with the closest propensity scores were selected sequentially based on the shortest Euclidean distance. Once matched, subjects were removed from subsequent matching rounds. This matching process continued until all possible triplets were matched, yielding a total of 79 matched sets comprising 237 eyes from 237 individuals. The data analyses were executed using Python (version 3.11) along with pandas (version 2.2.1) and scikit-learn (version 1.4.2) libraries.

### 4.3. Measurement of AGEs in the Fingertip Skin

At our institution, AGE measurement is routinely performed for ophthalmology patients, particularly for those attending the glaucoma clinic. To assess AGE accumulation, sAF was measured using an AGE Sensor (RQ-1101J-SET, Air Water Biodesign Inc., Kobe, Japan). The AGE scores were derived from fluorescence intensities at excitation and emission wavelengths of 365 nm and 440 nm, respectively. The resulting AGE scores positively correlate with levels of Nδ-(5-hydro-5-methyl-4-imidazolone-2-yl)-ornithine (MG-H1), a hyperglycemia-associated AGE [79]. Additionally, sAF correlates with collagen-linked fluorescence (CLF) measured from skin biopsies (excitation at 370 nm, emission at 440 nm), which represents both fluorescent and non-fluorescent AGEs, including pentosidine and MG-H1, and also correlates with other well-known AGEs such as Nε-(carboxymethyl)-lysine (CML) and Nε-(carboxyethyl)lysine [32,97,98]. Thus, sAF serves as a reliable surrogate marker for tissue AGE accumulation.

Measurements were taken from the middle finger of the non-dominant hand using a finger clip device to minimize melanin-related interference. Although venous autofluorescence intensity is approximately 1.5 times higher than that of skin, the fingertip area, which contains only capillaries and lacks veins, reduces potential venous fluorescence interference significantly [79]. All measurements were conducted by trained examiners. The AGE scores were recorded in arbitrary units. According to a previous pilot study, the coefficient of variation and the intraclass correlation coefficient (Cronbach’s α) for three consecutive AGE measurements were 6.7 ± 7.3% and 0.938, respectively [68].

### 4.4. Age Prediction from Fundus Photographs

For each participant, the “predicted age” was estimated using a computer algorithm based on color fundus photographs captured with a fundus camera (nonmyd WX, Kowa Company, Ltd., Aichi, Japan). All photographs had a 45° field of view and a resolution of 2144 pixels × 1424 pixels, saved in JPEG format. The algorithm used in this study was developed previously by our team [28]. Briefly, the model was initially trained using fundus images collected during routine health examinations. These training images were obtained using a non-mydriatic fundus camera (CR-DG 10, Canon, Tokyo, Japan) with a capture angle of 45°, resolution of 2400 × 1600 pixels, and stored in JPEG format. The training dataset included 10,679 images from 9433 eyes of 4929 non-glaucomatous participants. The mean age of subjects in the training dataset was 64.6 ± 12.8 years, with 47% males and 53% females. For algorithm modeling, we utilized the EfficientNet convolutional neural network model (“tf_efficientnet_b5.ns_jft_in1k”) as the foundation for predicting age. The AdaBelief optimizer was selected for training, and the deep learning models were implemented using Python (version 3.11.2) with PyTorch (version 2.01). Age predictions were generated using fundus images taken closest to the date of AGE measurement. “True age” was calculated from the participants’ date of birth and the date the photograph was taken. The “prediction difference” was calculated as predicted age minus true age. Thus, positive values indicated age acceleration, while negative values indicated age deceleration.

### 4.5. Statistical Analysis

Comparisons among the control, POAG, and EXG groups were conducted using one-way analysis of variance (ANOVA) or Fisher’s exact test for categorical variables across multiple groups. If a significant difference was identified among the three groups, post-hoc pairwise comparisons were performed using Tukey’s honest significant difference (HSD) test or Fisher’s exact test. A generalized linear regression model was utilized to control for the effects of age, sex, and other covariates. The variance inflation factor (VIF) was calculated to assess multicollinearity among explanatory variables. Correlations between AGE scores and age-related parameters were analyzed using Spearman’s rank correlation coefficient. All statistical procedures were conducted using JMP Pro software version 17.2 (SAS Institute, Inc., Cary, NC, USA). Statistical significance was determined at a *p*-value threshold of <0.05. English proofreading of the manuscript was assisted by ChatGPT version 4o (OpenAI, San Francisco, CA, USA).

## 5. Conclusions

This study revealed accelerated biological aging assessed by fundus photographs in patients with EXG. The accumulation of AGEs may serve as the biochemical basis underlying the accelerated ocular aging observed in fundus imaging.

## Figures and Tables

**Table 1:** Comparison of background parameters between three groups.

Parameter	Control		POAG		EXG		*p* Value ^a^
	Mean ± SD or N (%)	95% CI or N (%)	Mean ± SD or N (%)	95% CI or N (%)	Mean ± SD or N (%)	95% CI or N (%)	
N	79 eyes	79 subjects	79 eyes	79 subjects	79 eyes	79 subjects	-
Sex, N (%)	Male, 35 (44)	Female, 44 (56)	Male, 37 (47)	Female, 42 (53)	Male, 39 (49)	Female, 40 (51)	0.84
BMI, kg/m^2^	22.6 ± 3.8	21.8, 23.5	23.0 ± 3.4	22.2, 23.7	22.8 ± 2.9	22.1, 23.4	0.81
SBP, mmHg	141.4 ± 23.9	136.0, 146.7	144.7 ± 21.1	139.9, 149.4	149.4 ± 20.4	144.8, 154.0	0.07
DBP, mmHg	75.9 ± 14.2	72.8, 79.1	78.1 ± 12.8	75.2, 81.0	78.9 ± 13.3	75.9, 81.9	0.37
HR, cpm	76.1 ± 13.5	64.9, 67.8	74.1 ± 11.8	71.4, 76.7	76.0 ± 12.4	73.2, 78.8	0.51
Diabetes	Yes, 11 (14)	No, 68 (86)	Yes, 17 (22)	No, 62 (78)	Yes, 15 (19)	No, 64 (81)	0.50
BCVA, LogMAR	0.2 ± 0.2	0.1, 0.3	0.3 ± 0.5	0.2, 0.4	0.4 ± 0.6	0.2, 0.5	0.06
SERE, D	−0.7 ± 2.4	−1.3, −0.2	−2.1 ± 3.2	−2.8, −1.4	−1.4 ± 2.1	−1.8, −0.9	0.006 **
*p* Value ^b^, vs. Control	-		0.004 **		0.29		-
*p* Value ^b^, vs. POAG	-		-		0.20		-
Lens status	Phakia, 70 (89)	IOL, 9 (11)	Phakia, 31 (39)	IOL, 48 (61)	Phakia, 22 (28)	IOL, 57 (72)	<0.0001 **
*p* Value ^b^, vs. Control	-		<0.0001 **		< 0.0001 **		-
*p* Value ^b^, vs. POAG	-		-		0.18		-
IOP, mmHg	14.5 ± 2.9	13.9, 15.2	20.6 ± 8.5	18.7, 22.5	27.9 ± 11.7	25.3, 30.5	<0.0001 **
*p* Value ^b^, vs. Control	-		<0.0001 **		< 0.0001 **		-
*p* Value ^b^, vs. POAG	-		-		< 0.0001 **		-

^a^ *p* values are calculated between three groups by one-way analysis of variance for continuous variables and by Fisher’s exact probability test for categorical variables. ^b^ *p* values are calculated by post-hoc Tukey’s honest significant difference test for continuous variables and by pairwise Fisher’s exact probability test for categorical variables. Double asterisks (**) indicate *p*-values less than 0.01. POAG, primary open angle glaucoma; EXG, exfoliation glaucoma; SD, standard deviation; CI, confidence interval; BMI, body mass index; SBP, systolic blood pressure; DBP, diastolic blood pressure; HR, heart rate; cpm, counts per minute; BCVA, best-corrected visual acuity; LogMAR, logarithm of the minimum angle of resolution; SERE, spherical equivalent refractive error; D, diopter; IOP, intraocular pressure.

**Table 2:** Comparison of true age, predicted age, and prediction difference between three groups.

Parameter	Control		POAG		EXG		*p* Value ^a^
	Mean ± SD	95% CI	Mean ± SD	95% CI	Mean ± SD	95% CI	
True age, y	74.0 ± 11.1	71.5, 76.4	73.7 ± 10.1	71.4, 75.9	74.6 ± 8.8	72.6, 76.6	0.84
Predicted age, y	68.7 ± 0.8	67.0, 70.4	69.4 ± 7.8	67.6, 71.1	72.8 ± 0.8	71.3, 74.3	0.0005 **
*p* Value ^b^, vs. Control	-		0.82		0.0008 **		-
*p* Value ^b^, vs. POAG	-		-		0.006 **		-
Prediction difference, y	−5.3 ± 6.6	−7.7, −3.8	−4.3 ± 6.5	−5.8, −2.9	−1.8 ± 7.6	−3.5, −0.1	0.006 **
*p* Value ^b^, vs. Control	-		0.67		0.006 **		-
*p* Value ^b^, vs. POAG	-		-		0.06		-

^a^ *p* values are calculated between three groups by one-way analysis of variance. ^b^ *p* values are calculated by post-hoc Tukey’s honest significant difference test. Double asterisks (**) indicates *p*-values less than 0.01. Prediction difference = Predicted age − True age. POAG, primary open angle glaucoma; EXG, exfoliation glaucoma; SD, standard deviation; CI, confidence interval.

**Table 3:** Multivariate analysis of parameters associated with age prediction difference.

Parameter	Estimate	95% CI	*p* Value	VIF
True age, /y	−0.50	−0.57, −0.43	<0.0001 **	1.3
Sex, F/M	0.82	−0.45, 2.09	0.20	1.1
BMI, /cm/kg^2^	0.01	−0.17, 0.20	0.90	1.1
SBP, /mmHg	−0.03	−0.06,0.01	0.15	1.6
DBP, /mmHg	0.04	−0.02, 0.10	0.15	1.7
HR, /cpm	0.03	−0.02, 0.08	0.20	1.1
Diabetes, yes/no	0.71	−0.95, 2.36	0.40	1.1
BCVA, /logMAR	0.50	−1.00, 2.00	0.51	1.2
SERE, /D	0.05	−0.20, 0.31	0.67	1.2
Lens, IOL/phakia	1.98	0.44, 2.53	0.01 *	1.6
IOP, /mmHg	0.04	−0.04, 0.11	0.36	1.6
EXG/Control	2.13	0.19, 4.08	0.03 *	2.3
POAG/Control	−0.34	−2.09, 1.41	0.70	1.8

*p* values were calculated using a generalized linear regression model. A single asterisk (*) and double asterisks (**) indicate *p*-values less than 0.05 and 0.01, respectively. CI, confidence interval; VIF, variance inflation factor; BMI, body mass index; SBP, systolic blood pressure; DBP, diastolic blood pressure; HR, heart rate; cpm, counts per minute; BCVA, best-corrected visual acuity; LogMAR, logarithm of the minimum angle of resolution; SERE, spherical equivalent refractive error; D, diopter; IOP, intraocular pressure; POAG, primary open-angle glaucoma; EXG, exfoliation glaucoma.

**Table 4:** Comparison of predicted age and prediction difference between glaucoma and control groups across age tertiles.

	T1 (≤70.8 y)			T2 (70.8–79.2 y)			T3 (>79.2 y)		
	Control	POAG	EXG	Control	POAG	EXG	Control	POAG	EXG
N	26	27	26	26	26	27	27	26	26
Predicted age, y									
Mean ± SD	61.8 ± 8.4	64.4 ± 10.1	69.6 ± 6.7	70.6 ± 3.5	70.0 ± 3.6	74.6 ± 3.4	73.5 ± 4.0	73.9 ± 4.7	74.1 ± 3.4
95% CI	56.8, 59.7	61.1, 67.6	66.3, 73.0	69.2, 72.0	68.5, 71.4	73.2, 75.9	71.9, 75.1	72.1, 75.8	72.7, 75.5
*p* value	0.005 **			<0.0001 **			0.86		
Prediction difference, y									
Mean ± SD	−0.6 ± 6.5	1.3 ± 5.2	4.7 ± 5.6	−4.2 ± 3.4	−5.1 ± 3.8	−0.3 ± 4.3	−10.7 ± 5.1	−9.3 ± 5.1	−9.8 ± 4.2
95% CI	−3.3, 2.0	−0.7, 3.4	2.4, 6.9	−5.6, −2.9	−6.7, −3.6	−2.0, 1.4	−12.7, −8.7	−11.4, −7.3	−111.5, −8.1
*p* value	0.006 **			<0.0001 **			0.58		

*p* values are calculated between three groups in each tertile group by one-way analysis of variance. Double asterisks (**) indicates *p*-values less than 0.01. Prediction difference = Predicted age − True age. POAG, primary open angle glaucoma; EXG, exfoliation glaucoma; SD, standard deviation; CI, confidence interval.

**Table 5:** Correlations between AGE score and true age, predicted age, and prediction difference in subjects stratified by true age tertile.

	T1 (≤70.8 y)		T2 (70.8–79.2 y)		T3 (>79.2 y)	
	ρ	*p*-Value	ρ	*p*-Value	ρ	*p*-Value
True age, y	0.02	0.89	0.16	0.15	0.12	0.29
Predicted age, y	0.04	0.74	0.34	0.002 **	−0.04	0.70
Prediction difference, y	0.03	0.79	0.22	0.048 *	−0.09	0.41

All *p* values are calculated using Spearman’s rank correlation test. ρ represents Spearman’s rank correlation coefficient. The single asterisk (*) and double asterisks (**) indicate *p*-values less than 0.05 and 0.01, respectively. Prediction difference = Predicted age − True age.

## Data Availability

Data are fully available upon reasonable request to the corresponding author.

## Abbreviations

The following abbreviations are used in this manuscript:

AGEs	Advanced Glycation End products
BCVA	Best-Corrected Visual Acuity
BMI	Body Mass Index
CML	Carboxymethyl-Lysine
DBP	Diastolic Blood Pressure
ECM	Extracellular Matrix
EXG	Exfoliation Glaucoma
HR	Heart Rate
IOP	Intraocular Pressure
IOL	Intraocular Lens
MD	Mean Deviation
MGO	Methylglyoxal
OAG	Open-Angle Glaucoma
POAG	Primary Open-Angle Glaucoma
RGC	Retinal Ganglion Cell
RMSE	Root Mean Square Error
sAF	Skin Autofluorescence
SBP	Systolic Blood Pressure
SERE	Subjective Spherical Equivalent Refractive Error
T1, T2, T3	First, second, and third tertiles (age-based categories)
TM	Trabecular Meshwork
VIF	Variance Inflation Factor
